# Respiratory mechanics influence VO_2_ max in acute hypoxia in females

**DOI:** 10.1186/2046-7648-4-S1-A64

**Published:** 2015-09-14

**Authors:** Teruhiro Hanamaru, Tsuyoshi Yoshikawa, Takeshi Nishiyasu, Takeshi Ogawa

**Affiliations:** 1 Department of Physical and Health Education, Osaka Kyoiku University, Japan; 2 Institute of Health and Sports Science, University of Tsukuba, Japan

## Introduction

The magnitude of decrease in maximal oxygen uptake (VO_2_ max) in hypoxia (H) is more pronounced in male endurance athletes. In these subjects, high pulmonary ventilation (V_E_) could be beneficial in maintaining VO_2_ max in H [1]. Because females have smaller chest walls and narrower airways than males, V_E _during intensive exercise is mechanically limited [2]. Thus, it is hypothesised that in females, respiratory response and mechanics influenced the magnitude of decrease in VO_2_ max in H relative to males, despite lower VO_2_ max in females than in males. To test this hypothesis, we studied 22 healthy males and females as they performed an exhaustive cycling test in H and normoxia (N) conditions.

## Methods

Twenty-two healthy males (n = 12; VO_2_ max: 51 (7) ml.kg^-1^.min^-1^, age; 21 (2) yr, stature; 172 (3) cm, mass; 66 (6) kg) and females (n = 10; VO_2_ max = 44 (6) ml.kg^-1^.min^-1^, age; 21 (1) yr, stature; 159 (4) cm, mass; 53 (8) kg) performed the incremental cycle exercise test until exhaustion under N (20.9 % O_2_) and H (15.0 % O_2_) conditions. During the exercise test, we measured VO_2_ max and V_E_ max. To mechanically assess the respiratory work, we measured transpulmonary pressure by subtracting mouth pressure from esophageal pressure and calculated work of breathing (WOB) as the integrated area of the Ptp-volume loop.

## Results

The percentage decrease in VO_2_ max in H (% dVO_2_ max) tended to be larger in females than in males (−16% in males and −21% in females, p < 0.06). V_E_/VO_2_ was significantly (p < 0.05) higher in females than males, and it was significantly (p < 0.01) higher in H than in N in both genders. In females only, the % dVO_2_ max in H was significantly correlated to the extent of change in V_E _max (r = 0.79, p < 0.05). In comparison with N, WOB/V_E _in H tended to be lower in males (−13.1%) whereas it was 14.6% higher in females (not significant). Furthermore, in females, the % dVO_2_ max in H was significantly correlated to WOB/V_E _in H (r = −0.76, p < 0.05).

## Discussion

These results suggest that females have lower ventilatory mechanical efficiency than males, and VE is one of the factors causing this decrease in VO_2_ max in H. Further, there is a possibility that the oxygen demand at the respiratory muscles greatly increases against the increase in V_E _in H. Thus, high respiratory muscle work compromises blood flow to the active muscles [3], thereby limiting their peak work rate and VO_2_ max in H.

## Conclusion

Our findings demonstrated that in females, the respiratory muscle work efficiency affected the decrease in VO_2_ max in H, a decrease which tended to be larger in females than in males, despite the lower VO_2_ max in females compared with males.
